# Olfaction and Physical Function in Older Adults: Findings From Health ABC

**DOI:** 10.1093/geroni/igaa057.1710

**Published:** 2020-12-16

**Authors:** Yaqun Yuan, Zhehui Luo, Chenxi Li, Eleanor Simonsick, Eric Shiroma, Honglei Chen

**Affiliations:** 1 Michigan State University, East Lansing, Michigan, United States; 2 National Institute on Aging, Bethesda, Maryland, United States

## Abstract

The present study aims to investigate poor olfaction in relation to physical functioning in community-dwelling older adults and potential sex and race disparities. The analysis included 2511 participants aged 71-82 years (51.7% women and 38.4% blacks) from the Health Aging, and Body Composition (Health ABC) study. Olfaction was tested with the 12-item Brief Smell Identification Test (BSIT). Physical function measures included the Short Physical Performance Battery (SPPB), the Health ABC Physical Performance Battery (HABCPPB), gait speed of 20-meter walk, fast 400-meter walking time, grip strength, and knee extensor strength, repeatedly assessed annually or biennially for a follow-up of seven years. We analyzed each of these physical function measures using mixed models, adjusting for demographics, lifestyle, and comorbidities. For all measures except grip and knee extensor strength, poor olfaction was clearly associated with poorer physical performance at baseline and a faster decline over time. For example, at baseline, the multivariate adjusted SPPB was 8.23 ± 0.09 for participants with poor olfaction and 8.55 ± 0.09 for those with good olfaction (P = 0.02), after seven years of follow-up, the corresponding scores decreased to 6.46 ± 0.12 and 7.36 ± 0.10 respectively (cross-sectional P<0.001, and P for olfaction-by-year interaction < 0.001). For grip and knee extensor strength, similar differences were suggested but didn’t reach statistical significance. The overall results were similar by sex and race. In summary, poor olfaction is clearly associated with faster decline in physical functioning in older adults and future studies should investigate its potential health implications.

